# Relationship between gene regulation network structure and prediction accuracy in high dimensional regression

**DOI:** 10.1038/s41598-021-90791-6

**Published:** 2021-06-01

**Authors:** Yuichi Okinaga, Daisuke Kyogoku, Satoshi Kondo, Atsushi J. Nagano, Kei Hirose

**Affiliations:** 1 Graduate School of Mathematics, Kyushu University, 744 Motooka, Fukuoka, 819-0395 Japan; 2 The Museum of Nature and Human Activities, 6 Yayoigaoka, Sanda, Hyogo 669-1546 Japan; 3 Agriculture and Biotechnology Business Division, Toyota Motor Corporation, Miyoshi, Aichi 470-0201 Japan; 4 Faculty of Agriculture, Ryukoku University, Otsu, Shiga 520-2194 Japan; 5 Institute for Advanced Biosciences, Keio University, Tsuruoka, Yamagata 997-0017 Japan; 6 Institute of Mathematics for Industry, Kyushu University, 744 Motooka, Fukuoka, 819-0395 Japan; 7 RIKEN Center for Advanced Intelligence Project, 1-4-1 Nihonbashi, Chuo-ku, Tokyo, 103-0027 Japan

**Keywords:** Gene regulatory networks, Plant sciences

## Abstract

The least absolute shrinkage and selection operator (lasso) and principal component regression (PCR) are popular methods of estimating traits from high-dimensional omics data, such as transcriptomes. The prediction accuracy of these estimation methods is highly dependent on the covariance structure, which is characterized by gene regulation networks. However, the manner in which the structure of a gene regulation network together with the sample size affects prediction accuracy has not yet been sufficiently investigated. In this study, Monte Carlo simulations are conducted to investigate the prediction accuracy for several network structures under various sample sizes. When the gene regulation network is a random graph, a sufficiently large number of observations are required to ensure good prediction accuracy with the lasso. The PCR provided poor prediction accuracy regardless of the sample size. However, a real gene regulation network is likely to exhibit a scale-free structure. In such cases, the simulation indicates that a relatively small number of observations, such as $N=300$, is sufficient to allow the accurate prediction of traits from a transcriptome with the lasso.

## Introduction

Technological advancements have enabled the collection of highly multidimensional data from biological systems^1–4^. For example, RNA sequencing quantifies expression levels of thousands of genes. Such omics data is useful in predicting organismal traits, with empirical applications including diagnosis and classification of diseases and prediction of patient survival^5–8^ and possible future applications in predicting crop yields^9^, insecticide resistance^10^, and environmental adaptation^11^.

A common challenge in predicting traits from omics data is the dimension of the data far exceeding that of the sample size (known as high-dimensional regression). For example, if one is to apply least-squares estimation in multiple regression (e.g. ${\text{ trait }} \approx \beta _0 + \beta _1{\text{ gene}}_1 + \beta _2{\text{ gene}}_2 + \cdots$) to predict a trait value from a transcriptome, the sample size needs to be (at least) larger than the number of model parameters. However, because transcriptome studies typically observe thousands of genes, a sample size exceeding the number of genes is not realistic at present. In this case, high-dimensional regression modeling must be considered.

The least absolute shrinkage and selection operator (lasso^12^) is one of the most frequently used methods for high-dimensional regression. It simultaneously achieves variable selection and parameter estimation. Theoretically, the prediction accuracy of the lasso is highly dependent on the correlation structure among exploratory variables; it is high under certain strong conditions, such as the compatibility condition^13^. However, in practice, it is not easy to check whether the compatibility condition holds. Another popular estimation method for high-dimensional regression is principal component regression (PCR^14^). PCR is a two-stage procedure: first, principal component analysis is conducted for predictors, following which the regression model on which the principal components are used as predictors is fitted. This method may perform well when the exploratory variables are highly correlated.

It is reasonable to assume that gene regulation networks will result in conditional independence among the levels of gene expression^15–17^. Here, two variables are conditionally independent when they are independent given other variables (e.g. two focal variables are independently influenced by a third variable^18^). When a random vector of exploratory variables follows a multivariate normal distribution, two variables are conditionally independent if and only if the corresponding element of the inverse covariance matrix is zero. Essentially, the networks are characterized by the nonzero pattern of the inverse covariance matrix.

One of the most notable characteristics of biological networks is their scale-free nature, that is, the degree distribution of the network follows a power-law expressed as $p(x) \propto x^{-\gamma }$ ($\gamma > 1$)^19,20^. Empirical studies suggest that biological networks are often scale-free^21–23^, although exceptions have also been found^24^. Therefore, it is reasonable to consider the problem of high-dimensional regression when the networks of exploratory variables are scale-free. Here, it should be noted that the relative performance of different high-dimensional regression techniques may depend on sample sizes. However, to the best of our knowledge, the effect of the gene regulation network structure together with sample size on prediction accuracy has not yet been sufficiently investigated.

This paper provides a general simulation framework to study the effects of correlation structure in explanatory variables. As an example, the prediction of ambient temperature from the transcriptome, for which good empirical data is available^11,25^, is considered. It should be noted that the implementation of the proposed procedure is independent of the empirical data^11,25^; the proposed framework may be applied to predict any consequence of gene expression differences. The proposed framework is based on the Monte Carlo simulations. Three datasets of transcriptome and their traits are generated. The datasets are characterized by the covariance structure of exploratory variables; one of the covariance structures corresponds to the scale-free gene regulation network. Both lasso and PCR are applied to these simulated datasets to investigate the prediction accuracy with different types of gene regulation networks. The sample size is also varied to examine its effect on the prediction accuracy.

The remainder of this paper is organized as follows. Section “Prediction methods for high-dimensional data” describes prediction methods for high-dimensional regression in the given simulation. Section “Simulation framework” discusses the proposed simulation framework. Finally, Section “Concluding remarks” presents the concluding remarks.

## Prediction methods for high-dimensional data

Suppose that we have *n* observations $\{(\varvec{x}_{i}, y_{i})\mid i=1,\ldots ,n\},$ where $\varvec{x}_{i}$ are *p*-dimensional vector of explanatory variables and $y_{i}$ are responses $(i=1,\ldots ,n)$. Let $X = (\varvec{x}_{1}, \ldots , \varvec{x}_{n})^{T}$ and $\varvec{Y} = (y_{1}, \ldots , y_{n})^{T}$. Consider the linear regression model:

$$\begin{aligned} \varvec{Y } = X \varvec{\beta } + \varvec{\epsilon }, \end{aligned}$$

where $\varvec{\epsilon } = (\epsilon _{1}, \ldots , \epsilon _{n})^{T}$ is a vector of error variables with $E(\varvec{\epsilon }) = \varvec{0}$ and ${V}(\varvec{\epsilon }) = \sigma ^{2} I_{n}$.

### Lasso

The lasso minimizes a loss function that consists of quadratic loss with a penalty based on an $L_1$ norm of a parameter vector:

1 $$\begin{aligned} \hat{\varvec{\beta }} = \mathop {{\text{arg}~\text{min}}}\limits _{\varvec{\beta }} \frac{1}{2}\Vert \varvec{Y}-X\varvec{\beta }\Vert_2 ^2 + \lambda \Vert \varvec{\beta }\Vert _{1}, \end{aligned}$$

where $\lambda > 0$ is a regularization parameter. Because of the nature of the $L_1$ norm in the penalty term, some of the elements of the coefficients are estimated to be exactly zero. Thus, variable selection is conducted, and only variables that correspond to nonzero coefficients affect the responses.

### PCR

In some cases, the first few largest eigenvalues of the covariance matrix of predictors (i.e., proportional contributions of principle components) can be considerably large (e.g., spiked covariance model^26^). In such a case, the lasso may not function effectively in terms of both prediction accuracy and consistency in model selection, because the conditions for its effective performance (e.g., compatibility condition^27^) may not be satisfied. This issue could be addressed using PCR because it transforms data with a large number of highly correlated variables into a few principal components. In the first stage of PCR, principal component analysis is applied to predictors. The *i*th observation of predictor, $\varvec{x}_i$, is linearly mapped onto a $d \ (<p)$-dimensional vector, $\varvec{z}_i = A^{T}\varvec{x}_i$, where *A* is a $p \times d$ matrix. The matrix *A* is obtained by the following least squares optimization problem^28^:

$$\begin{aligned} A = \mathop {{\text{arg}~\text{min}}}\limits _A \sum _{i=1}^n \Vert (\varvec{x}_i - \bar{\varvec{x}}) - AA^T(\varvec{x}_i - \bar{\varvec{x}}) \Vert_2 ^2 \quad {\text {subject to}} \quad A^TA=I_d. \end{aligned}$$

here, $\bar{\varvec{x}}$ is the sample mean vector, that is, $\bar{\varvec{x}}=\sum _{i=1}^n\varvec{x}_i/n$. In this work, the number of projected dimensions, *d*, was chosen such that *d* principle components collectively explain 90% or more variance (and $d-1$ principle components do not). Then, in the second stage, regression analysis is conducted, for which the principal components, ${\{\varvec{z}_1,\ldots ,\varvec{z}_n \}}$, are used as predictors. Here, the regression coefficients in the second stage are estimated by the lasso.

## Simulation framework

**Figure 1:**
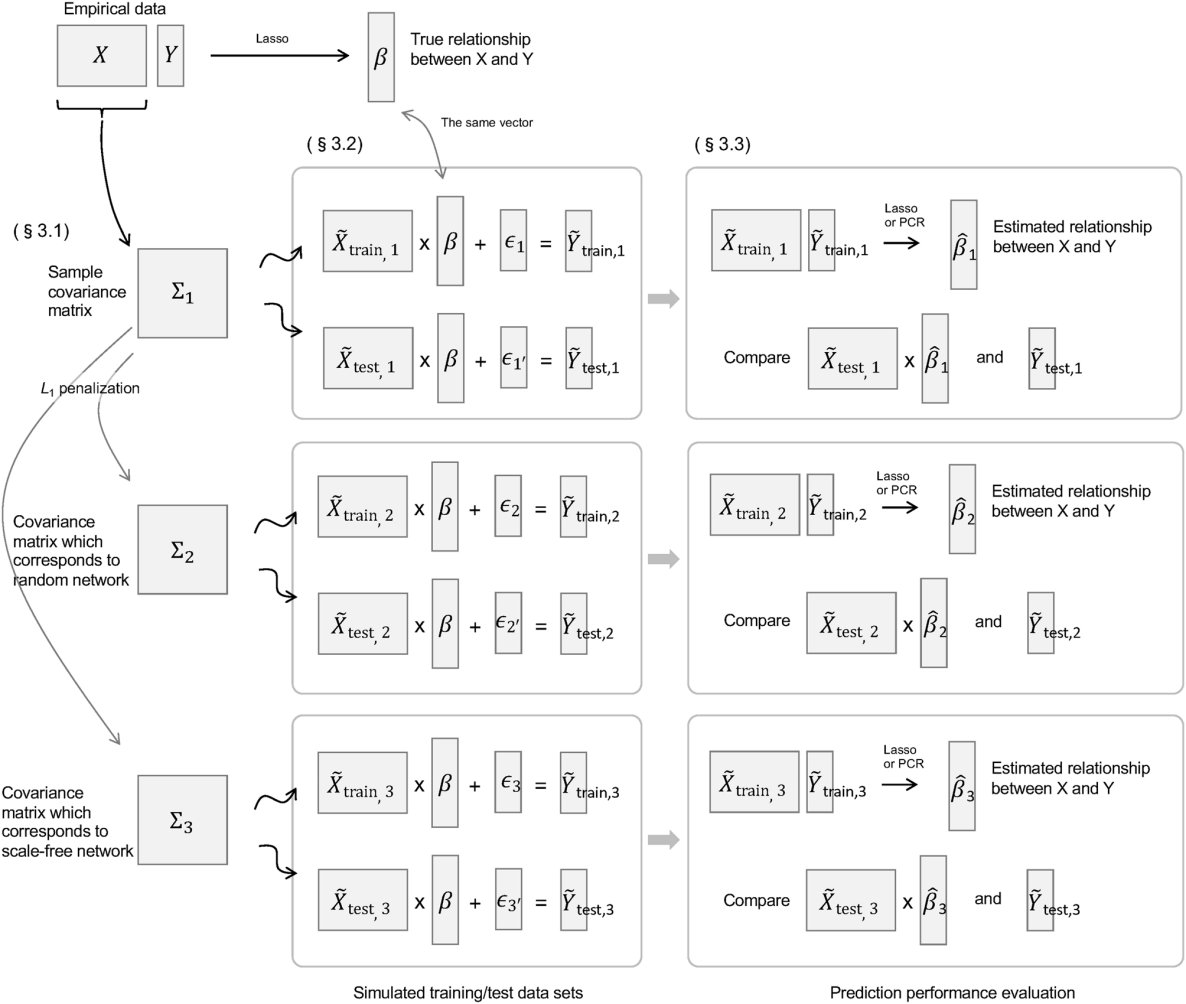
Overview of the simulation.

An overview of the simulation is presented in Fig. 1. First, the model that defines the relationship between the trait and the levels of gene expression was parameterized. This was done using the empirical data^11^, which quantified the transcriptome of wild *Arabidopsis halleri* subsp. *gemmifera* weekly for two years in their natural habitat as well as bihourly on the equinoxes and solstices (*p* = 17,205 genes for $n=835$ observations). Three types of simulated data were generated using different covariance matrices of genes, denoted as $\Sigma _{j}$ ($j = 1,2,3$). $\Sigma _{1}$ is the sample covariance matrix of genes. Generally, none of the elements of the inverse of sample covariance matrix are exactly zero, implying that each gene interacts with all the other genes. Such a fully connected network is ineffective in terms of interpretation of the mechanism of gene regulation. Thus, two other covariance matrices were produced to simulate sparse networks based on the sample covariance matrix $\Sigma _{1}$. $\Sigma _{2}$ is generated by the graphical lasso^29^, which corresponds to the random graph. Although the graphical lasso is widely used because of its computational efficiency, real networks are often scale-free. Therefore, $\Sigma _{3}$, which corresponds to the scale-free network, was generated here. The estimation of scale-free networks is achieved by the reweighted graphical lasso^30^. Based on these three covariance matrices $\Sigma _{j}$ ($j = 1,2,3$), the simulated transcriptome data were generated from the multivariate normal distribution. The simulated trait data were generated from simulated transcriptome data. Finally, lasso and PCR were applied to these simulated data to compare their prediction accuracies. The sample sizes of the simulated data were varied to investigate the relationship between prediction accuracy and sample sizes.

### Evaluation of the estimation procedure

The performance of the estimation procedure is investigated by the following expected prediction error:

$$\begin{aligned} E\left[ \left\| \varvec{Y}^{*}-(X^{*})^{T}\hat{\varvec{\beta }} \right\|_2^2\right] , \end{aligned}$$

where $X^{*}$ and $\varvec{Y}^{*}$ follow $X^* \sim N(\varvec{0},\Sigma _j)$ $(j = 1,2,$ or 3) and $\varvec{Y}^* \sim N((X^{*})^{T}\varvec{\beta },\sigma ^2I_n)$, respectively. The estimator $\hat{\varvec{\beta }}$ is obtained using current observations, while $X^{*}$ and $\varvec{Y}^{*}$ correspond to future observations. The $\Sigma _j$ ($j = 1,2,3$), $\varvec{\beta }$, and $\sigma ^2$ are true values but unknown. In practice, these parameters are defined by using the actual dataset, (*X*, ***Y***). Detail of setting of these parameters will be presented in the next subsection.

To estimate the expected prediction error, the Monte Carlo simulation is conducted. We first randomly generate training and test data, $(\tilde{~X}_{train}, \tilde{~\varvec{Y}}_{train})$ and $(\tilde{~X}_{test}, \tilde{~\varvec{Y}}_{test})$, respectively. Here, $\tilde{~X}_{train}$ follows a multivariate normal distribution with mean vector $\mu _X$ and variance–covariance matrix $\Sigma _j$, where $\mu _X$ is the sample mean of *X*. Then, $\tilde{~\varvec{Y}}_{train}$ is generated by $\tilde{~\varvec{Y}}_{train} = \tilde{~X}_{train} \varvec{\beta } + \varvec{\epsilon }$, where $\varvec{\epsilon }$ is a random sample from $N(\varvec{0}, \sigma ^2I)$ with *I* being an identity matrix. The test data, $(\tilde{~X}_{test}, \tilde{~\varvec{Y}}_{test})$, are generated by the same procedure as $(\tilde{~X}_{train}, \tilde{~\varvec{Y}}_{train})$ but independent of $(\tilde{~X}_{train}, \tilde{~\varvec{Y}}_{train})$. The number of observations for the training and test data are *N* ($N = 50, 100, 200, 300, 500, 1000$) and 1000, respectively. The lasso and the PCR are performed with training data $(\tilde{~X}_{train}, \tilde{~\varvec{Y}}_{train})$, following which RMSE is calculated in (10). The above process, from random generation of data to RMSE calculation, was performed 100 times.

### Parameter setting

#### Covariance structures

Here, the characterization of the network structure of predictors by conditional independence is considered. When the predictors follow a multivariate normal distribution, the network structure based on the conditional independence corresponds to the nonzero pattern of the inverse covariance (precision) matrix. In other words, the network structure is characterized by the inverse covariance matrix of predictors.

Let *S* be the sample covariance matrix of predictors, that is, $S = \sum _{i = 1}^n(\varvec{x}_i-\bar{\varvec{x}})(\varvec{x}_i-\bar{\varvec{x}})^T/n$. Let $\Omega _j = \Sigma _j^{-1}$ $(j = 1,2,3)$. $\Sigma _{1}$ is a ridge estimator of the sample variance-covariance matrix, that is, $\Sigma _1 = S + \delta I$. Here $\delta$ is a small positive value (in this simulation, $\delta = 10^{-5}$). The term $\delta I$ allows the existence of $\Omega _1$. Note that because $\Omega _1$ is not sparse, it leads to the complete graph, which is of no use in interpreting gene regulatory networks. To generate a covariance matrix whose inverse matrix is sparse, $L_1$ penalization is employed for the estimation of $\Omega _2$ and $\Omega _3$ as follows:

2 $$\begin{aligned} \hat{~\Omega~}_j = \mathop {\text{arg}~\text{max}}\limits _{\Omega } \left\{ \log |\Omega | - {\text{tr}}(\Omega S) - P_j(\Omega )\right\} \quad (j = 2,3), \end{aligned}$$

where $P_j(\Omega )$ $(j = 2,3)$ are penalty terms which enhance the sparsity of the inverse covariance matrix. To estimate the sparse inverse covariance matrix, the lasso penalty is typically used as follows:

3 $$\begin{aligned} P_2(\Omega ) = \rho \sum _{i = 1}^p \Vert \varvec{\omega }_{(-i,\cdot )}\Vert _1, \end{aligned}$$

where $\varvec{\omega }_{(-i,\cdot )} = (\omega _{i1},\omega _{i2},\ldots ,\omega _{i(i-1)},\omega _{i(i + 1)},\ldots ,\omega _{ip})^T \in \mathbb {R}^{p-1}$. The problem (3) is referred to as the graphical lasso^29^, and there exists several efficient algorithms to obtain the solution^31–33^. The estimator of (2) with (3) corresponds to $\Omega _2$ and $\Sigma _2 = \Omega _2^{-1}$.

The lasso penalty (3) does not enhance scale-free networks. It penalizes all edges equally so that the estimated graph is likely to be a random graph, that is, the degree distribution becomes a binomial distribution. To enhance scale-free networks (i.e., power-law distribution), the log penalty^30^ is used as follows:

4 $$\begin{aligned} P_3(\Omega ) = \frac{\rho }{2} \sum _{i=1}^p \left\{ \log \left( \Vert \varvec{\omega }_{(-i,\cdot )} \Vert _1 + a_i \right) + \log \left( \Vert \varvec{\omega }_{(\cdot ,-i)} \Vert _1 + a_i \right) \right\} , \end{aligned}$$

where $\varvec{\omega }_{(\cdot ,-i)} = (\omega _{1i},\omega _{2i},\ldots ,\omega _{(i-1)i},\omega _{(i + 1)i},\ldots ,\omega _{pi})^T$ and $a_i > 0$ are tuning parameters. We note that the penalty (4) is slightly different from original definition^30^, expressed as

5 $$\begin{aligned} P(\Omega ) = \rho \sum _{i=1}^p \log \left( \Vert \varvec{\omega }_{(-i,\cdot )} \Vert _1 + a_i \right) . \end{aligned}$$

When we do not assume that $\omega _{ij} = \omega _{ji}$, the estimate of the inverse covariance matrix with (5) is not symmetric. Since the original graphical lasso algorithm does not assume that $\omega _{ij} = \omega _{ji}$^31,34^, we slightly modify the penalty as in Eq. (4). Notably, $P_3(\Omega )$ in (4) coincides with (5) when $\omega _{ij} = \omega _{ji}$. From a Bayesian viewpoint, the prior distribution which corresponds to the log penalty becomes the power–law distribution^30^; thus, the penalty (4) is likely to estimate the scale-free networks. The estimator of (2) with (4) corresponds to $\Omega _3$.

Because the log-penalty (4) is nonconvex, it is not easy to directly optimize (2). To implement the maximization problem (2), the minorize-maximization (MM) algorithm^35^ has been constructed^30^, in which the weighted lasso penalty $P_M^{(t)}(\Omega )$ with current parameter $\Omega _3^{(t)}$ is used:

6 $$\begin{aligned} P_M^{(t)}(\Omega ) = \sum _{i = 1}^p\sum _{j \ne i}\rho _{ij}^{(t)}|\omega _{ij}|, \end{aligned}$$

where $\rho _{ij}^{(t)}$ are the weights

7 $$\begin{aligned} \rho _{ij}^{(t)} = \frac{1}{2} \left( \frac{\rho }{ \Vert \varvec{\omega }_{(-i,\cdot )}^{(t)} \Vert _1 + a_i} + \frac{\rho }{ \Vert \varvec{\omega }_{(\cdot ,-j)}^{(t)} \Vert _1 + a_j} \right) . \end{aligned}$$

In general, $\hat{~\Omega~}$ must be symmetric, so that Eq. (7) can be expressed as

8 $$\begin{aligned} \rho _{ij}^{(t)} = \frac{1}{2}\left( \frac{\rho }{ \Vert \varvec{\omega }_{(-i,\cdot )}^{(t)} \Vert _1 + a_i} + \frac{\rho }{ \Vert \varvec{\omega }_{(-j,\cdot )}^{(t)} \Vert _1 + a_j}\right) . \end{aligned}$$

Because the weighted graphical lasso can be implemented by a standard graphical lasso algorithm, the estimator is obtained as the following algorithm.

1. Set $t = 0$. Get $\Omega _3^{(0)}$ via ordinary graphical lasso. Repeat 2 to 4 until convergence.
2. Update weights $\rho _{ij}^{(t)}$ using (7).
3. Get $\Omega _3^{(t+1)}$ via the weighted graphical lasso (2) with penalty (6).
4. $$t \leftarrow t+ 1$$.

To obtain $\Sigma _2$ and $\Sigma _3$, the tuning parameters $a_i$ $(i = 1\dots ,p)$ and $\rho$ must be determined. Following the experiments^30^, $a_i = 1$ was set for $i = 1\dots ,p$. To select the value of the regularization parameter $\rho$, several candidates were first prepared. In our simulation, the candidates were $\rho = 0.3,0.4,0.5,0.6,0.7$. From these, the value of $\rho$ was selected such that the extended Bayesian information criterion (EBIC^36,37^)

9 $$\begin{aligned} {\text{EBIC}}= -n\left\{ \log |\Omega _2| - {\text{tr}}(\Omega _2S) \right\} + q\log n + 4 q \delta \log p \end{aligned}$$

was minimized. Here, *q* is the number of nonzero parameters of the upper triangular matrix of $\hat{~\Omega~}$, and $\delta \in [0,1)$ is a tuning parameter. As the value of $\delta$ increases, a sparser graph is generated. Note that $\delta = 0$ corresponds to the ordinary BIC^38^. We set $\delta = 0.5$ because $\delta = 0.5$ is shown to yield good performance in both simulated and real data analyses^37^. As a result, the EBIC selected $\rho = 0.5$.

The upper triangular matrix $\Omega _3$ must be estimated with the reweighted graphical lasso problem. A value of *p* = 17205 results in $p(p+1)/2 \approx 148$ million parameters. As a result, with the machine used in this study (Intel Core Xeon 3 GHz, 128 GB memory), it would take several days to conduct the reweighted graphical lasso approach, even with a small number of iterations such as $T = 5$. For this reason, $T = 5$ iterations were employed to produce $\Sigma _3$ here. Finally, $\Sigma _2$ and $\Sigma _3$ were scaled such that their signal-to-noise ratio became $\Sigma _1$.

**Figure 2:**
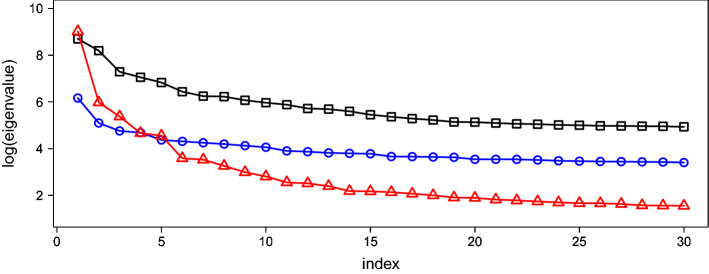
Logarithm graph of the largest 30 eigenvalues of $\Sigma _{1}$ (square), $\Sigma _{2}$ (circle) and $\Sigma _{3}$ (triangle). The horizontal axis expresses the index of eigenvalues arranged in descending order.

Figure 2 depicts the logarithm of the largest 30 eigenvalues of $\Sigma _{j}$ ($j = 1, 2, 3$). The first few largest eigenvalues of $\Sigma _{3}$ are significantly larger than those of $\Sigma _{2}$, implying that the scale-free networks tend to produce predictors with large correlations.

#### Regression parameters

The values of $\varvec{\beta }$ and $\sigma ^2$ are determined as follows. First, 10-fold cross-validation is performed as described below, and the regularization parameter $\lambda$ in (1) is selected. The data (*X*, *Y*) are divided into ten datasets, $(X^{(j)},\varvec{Y}^{(j)})$ $(j = 1,\ldots ,10)$, which consist of almost equal sample sizes. Let $X^{(-j)}=(X^{(1)},\ldots ,X^{(j - 1)},X^{(j + 1)},\ldots ,X^{(10)})$, and $\varvec{Y}^{(-j)}=(\varvec{Y}^{(1)},\ldots ,\varvec{Y}^{(j - 1)},\varvec{Y}^{(j + 1)},\ldots ,\varvec{Y}^{(10)})$ ($j = 1,\ldots ,10$). For each *j* ($j = 1,\ldots ,10$), the training and test data are defined by $(X^{(-j)},\varvec{Y}^{(-j)})$ and $(X^{(j)},\varvec{Y}^{(j)})$, respectively. Then, the parameter $\hat{\varvec{\beta }}^{(j)}$ ($j=1,\ldots ,10$) is found by the lasso:

$$\begin{aligned} \hat{\varvec{\beta }}^{(j)} = \mathop {{\text{arg}~\text{min}}}\limits _{\varvec{\beta }} \left( \Vert \varvec{Y}^{(-j)} - X^{(-j)} \varvec{\beta }\Vert _{2}^{2} + \lambda \Vert \varvec{\beta }\Vert _{1}\right) . \end{aligned}$$

For each *j* ($j=1,\ldots ,10$), the verification error is calculated as follows:

$$\begin{aligned} {\text{CV}}^{(j)} = \frac{1}{\# \varvec{Y}^{(j)}} \Vert \varvec{Y}^{(j)} - X^{(j)} \hat{\varvec{\beta }}^{(j)}\Vert _{2}^{2}. \end{aligned}$$

**Figure 3:**
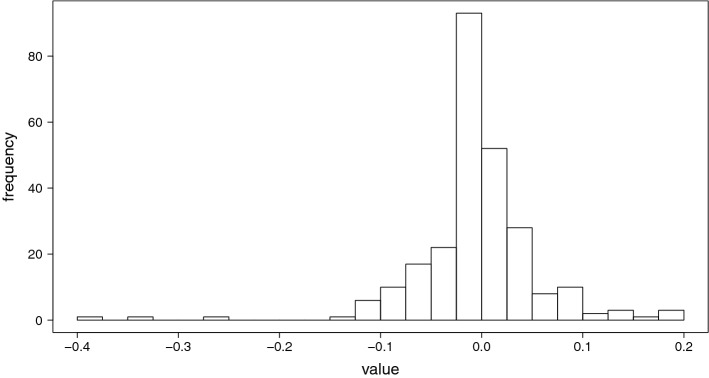
Histogram of 259 nonzero parameters of $\varvec{\beta }$.

Then, $\lambda$ is adopted such that it minimizes ${\text{CV}} = \frac{1}{10} \sum _{j = 1}^{10} {\text{CV}}^{(j)}$, the mean of ${\text{CV}}^{(j)}$. Following this, the dataset (*X*, *Y*) is again randomly divided into two datasets: test data $(X_{test}, \varvec{Y}_{test})$ and training data $(X_{train}, \varvec{Y}_{train})$. Lasso estimation (1) is performed using the training data, with $\lambda$ obtained by the above 10-fold cross-validation. Then, $\varvec{\beta }$ is defined as the lasso estimator, resulting in the number of nonzero parameters of $\varvec{\beta }$ being 259. Figure 3 shows the histogram of nonzero parameters of $\varvec{\beta }$. It is seen that the majority of the nonzero coefficients were close to zero; only 15 parameters had absolute values larger than 0.1.

In addition, the root mean squared error (RMSE) is calculated as follows:

10 $$\begin{aligned} {\text{ RMSE }} = \frac{1}{\sqrt{\# \varvec{Y}_{test}}} \Vert \varvec{Y}_{test} - X_{test}\hat{\varvec{\beta }}\Vert _{2}, \end{aligned}$$

and the variance of errors, $\sigma ^{2}$, is defined by $\sigma ^{2} = ({\text{RMSE}})^{2}$.

## Results

**Figure 4:**
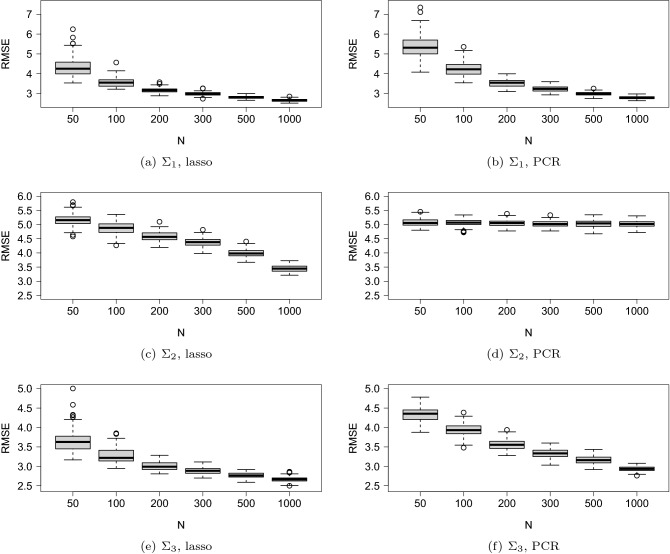
Box and whisker plot of RMSE. The variance-covariance matrix used in the simulations is $\Sigma _{1}$ in (**a**, **b**), $\Sigma _{2}$ in (**c**, **d**), and $\Sigma _{3}$ in (**e**, **f**). The regression model is estimated by the lasso in (**a**), (**c**), and (**e**) and by PCR in (**b**), (**d**), and (**f**).

**Figure 5:**
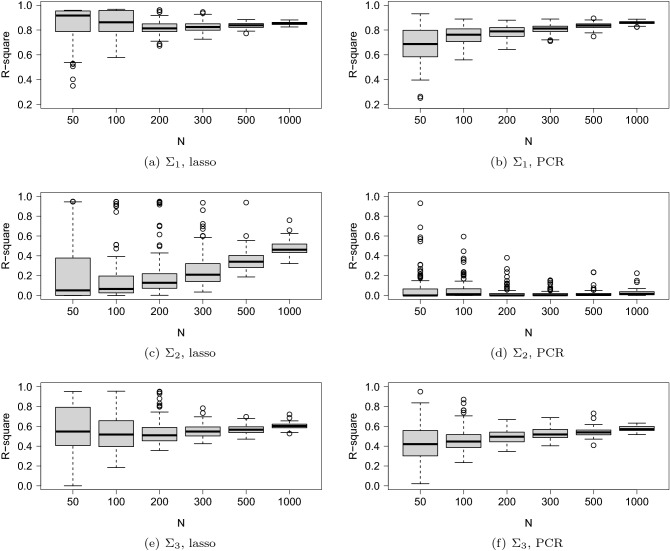
Box and whisker plot of $R^2$. The variance-covariance matrix used in the simulations is $\Sigma _{1}$ in (**a**, **b**), $\Sigma _{2}$ in (**c**, **d**), and $\Sigma _{3}$ in (**e**, **f**). The regression model is estimated by the lasso in (**a**), (**c**), and (**e**) and by PCR in (**b**), (**d**), and (**f**).

The box and whisker plot of the RMSE and the coefficient of determination ($R^2$) are illustrated in Figs. 4 and 5. The horizontal axis is *N* (the number of observations of training data) and the vertical axis is the RMSE or $R^2$ based on 1000 observations of test data.

**Figure 6:**
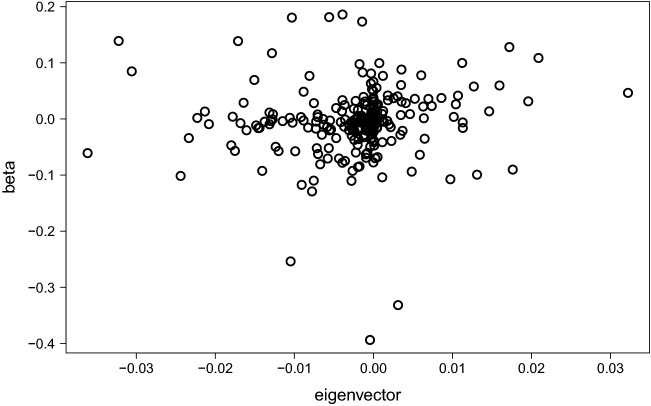
Scatter plot of $\varvec{\beta }$ and the eigenvector corresponding to the maximum eigenvalue of $\Sigma _2$. The nonzero elements of $\varvec{\beta }$ are not drawn.

We compared the performance of the lasso with that of the PCR. When $\Sigma _1$ and $\Sigma _3$ were used, the PCR performed worse than the lasso for small sample sizes. For $\Sigma _{2}$, the prediction performance with PCR was unsatisfactory even when the sample size *N* increased. The poor performance of the PCR can be attributed to the predictors associated with small eigenvalues; these predictors affected the prediction performance. Figure 6 depicts a scatter plot of nonzero elements of $\varvec{\beta }$ and the eigenvector for the maximum eigenvalue of $\Sigma _2$. As can be seen, only a significant amount of correlation existed; in fact, the correlation coefficient was only 0.068.

The prediction accuracy was compared among the three covariance structures. In all the cases except PCR with $\Sigma _2$, the values of RMSE decreased and $R^2$ increased with the increase in the value of *N*. Further, $R^2$ was unstable for small sample sizes for all the cases when the lasso was applied. For large sample sizes, the $R^2$ of $\Sigma _1$ was better than that of $\Sigma _2$ and $\Sigma _3$. As described before, $\Sigma _1$ was the sample covariance matrix, while $\Sigma _3$ (and $\Sigma _2$) was estimated using the graphical lasso. As the lasso-type regularization methods shrink parameters toward zero, the correlations among the exploratory variables reduce when the graphical lasso is used. Therefore, $\Sigma _2$ and $\Sigma _3$ resulted in smaller correlations as compared to $\Sigma _1$. Consequently, the $R^2$ may increase with stronger correlations. We compared the RMSE results of $\Sigma _2$ and $\Sigma _3$. With $\Sigma _2$, we found that a sufficiently large number of observations is required to yield a small RMSE with the lasso. Meanwhile, $\Sigma _3$ resulted in a small RMSE with a relatively small number of observations, such as $N=300$.

### Code availability

The proposed simulation is implemented in R package simrnet, which is available at https://github.com/keihirose/simrnet. Below is a sample code of the simrnet in R: 
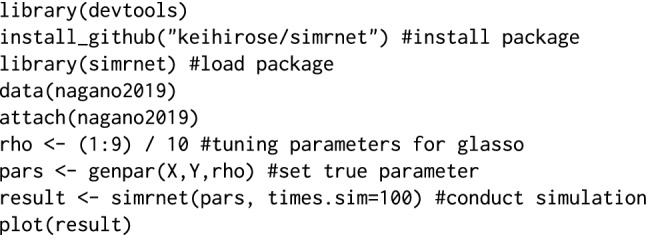


When $p = 100$, it took less than 12 min to conduct the simulation with 100 replications using the machine employed herein (Intel Core Xeon 3 GHz, 128  GB memory). For high-dimensional data such as *p* = 17,205, which was used in the simulation presented in this paper, several days were required to complete the simulation task.

## Concluding remarks

In a gene regulation network, a gene regulates a small portion of a genome, not all the genes in a genome. This indicates that gene regulation network is expected to be a sparse network rather than a complete graph. Therefore, two covariance matrices indicating sparse networks ($\Sigma _{2}$, $\Sigma _{3}$) were prepared in addition to a covariance matrix derived from empirical data ($\Sigma _{1}$). Generally, although hundreds of genes contribute to defining a trait, their contributions are not equal. It is frequently observed that genes regulating a trait include a few large-effect genes and many small-effect genes. This property was reflected in the distribution of $\varvec{\beta }$ (Fig. 3). We considered the case where a limited number of regression coefficients significantly contributed to the definition of a trait. The Monte Carlo simulation result indicated that regardless of the network structure, the number of observations should be greater than at least 200 to accurately predict traits from a transcriptome ($\Sigma _{1}$, $\Sigma _{3}$, Figs. 4 and 5). We also found that the lasso generally provided better accuracy than the PCR. In particular, when the gene regulation network was random ($\Sigma _{2}$), the prediction accuracy of the PCR was poor even if the sample size increased. In conclusion, it is important to sufficiently secure large sample sizes when performing regression analysis of data that exhibits either the random graph and the scale-free network. Additionally, we concluded that the lasso would be preferable to the PCR to ensure a good prediction accuracy.

Conventional theory on the relationship between RMSE and sample size has been developed under the assumption that the sample size exceeds the number of exploratory variables^39^. However, omics data, which is rapidly being accumulated, results in high dimensional data with strong correlations. Thus, our simulation study considered more complicated settings than the traditional ones. Our simulation, or its extension, may be used in the future to find clues about theoretical aspects that may ultimately lead to the development of a sample size determination technique for omics data.

Other than the scale-free network, the small-world network is another notable property in the networks literature^40^. The definition of the small-word networks is that the shortest path length between two randomly chosen variables is proportional to $\log p$; that is, it is considerably small compared with the network size. The small-world networks have been investigated in various fields of research, including the biology^41–43^. Some statistical properties of the small-world networks have also been studied^44–46^. The investigation of the prediction accuracy in the small-world networks would be interesting but beyond the scope of this research. We would like to take this as a future research topic. The development of methods that provides better prediction accuracy than the lasso in various network structures with small sample sizes would also be an important future research topic.
